# Enhanced health evaluation in mice using continuous home-cage monitoring and machine learning: a multicentric study

**DOI:** 10.1038/s41684-026-01745-2

**Published:** 2026-05-28

**Authors:** Jeetendra Eswaraka, Céline Gommet, Dimitri Diomaiuta, Mara Rigamonti, Giorgio Rosati, Stefano Gaburro, Michael Zwick, Laurent Bégoud, Xavier Warot, Raphaël Doenlen

**Affiliations:** 1 Rutgers, The State University of New Jersey, Piscataway, NJ USA; 2 Sanofi, Vitry-sur-Seine, France; 3 Tecniplast Spa, Buguggiate, Italy; 4 Center of PhenoGenomics, School of Life Sciences, Ecole Polytechnique Fédérale de Lausanne (EPFL), Lausanne, Switzerland

**Keywords:** Machine learning, Physiology

## Abstract

Ensuring the health of laboratory rodents is critical for ethical research and maintaining scientific integrity. Traditional daily visual observations by trained technicians, conducted during the rodents’ sleep period, often fail to detect subtle but critical health indicators due to the short duration of inspections and obstructions from enrichment materials. Here we aimed to improve health checks in mice by utilizing continuous home-cage monitoring coupled with machine learning (ML) algorithms. We hypothesized that reduced locomotion in mice would indicate distress or sickness, and that continuous tracking would identify clinical cases earlier than visual checks. We retrospectively analyzed locomotion data from three institutions using the same sensor technology and applied ML/artificial intelligence (AI) models to generate digital alerts for potential clinical cases. These alerts were then compared with clinical records to verify the accuracy of the predictions. Our results demonstrated that the ML algorithm identified animals in distress –3 to –6 days before verifiable clinical signs or death were noticed, with an accuracy of 66–80% on day –3 and 80–91% on day −6. This indicates that continuous monitoring of animal locomotion is a superior predictor of animal health compared with human observation. The findings suggest that augmenting visual checks with AI modeling can greatly improve animal welfare by identifying subclinical cases, enhancing study endpoints, increasing the rigor and reproducibility of research, and improving operational efficiency. Our work underscores the potential of integrating advanced monitoring systems and AI in laboratory animal facilities, marking a substantial step forward in the field of animal welfare and research methodology.

## Abstract (web summary)

This study shows that continuous home-cage monitoring with machine learning detects distress in mice days earlier than visual checks, improving animal welfare, study rigor and efficiency through early identification of subclinical illness.

## Main

Ensuring the health of laboratory rodents daily is essential for ethical research and maintaining scientific integrity. Accurate knowledge of the health status of experimental animals is pivotal to high scientific and ethical standards in biomedical research^1^. This daily observation is mandated by regulations^2–5^ and is typically performed through visual inspections during the daytime to suit the needs of human observers^6^. However, these methods often fail to detect subtle but critical health indicators owing to the short duration of inspections and obstructions from enrichment materials in the cages that hinder clear visual observation. Mice, being nocturnal, are inactive during this period of observation, which further limits the effectiveness of these checks^7^.

Home-cage monitoring (HCM) technologies are used in behavioral phenotyping research. These technologies can quantify animal locomotor activity, using automated video analysis^8^ capabilities, and/or radiofrequency identification transponders/readers or sensors beneath the cage^9–11^. These systems, by continuously detecting physiological and behavioral data with minimal human interference, can detect disruptions in natural animal behaviors and, therefore, have the potential to exceed the capabilities of manual inspections currently used in animal facilities^12^. Automated HCM is a non-invasive, reliable mode of health surveillance allowing caretakers to more efficiently detect and respond to early signs of illness in laboratory rodent populations^12^.

Machine learning (ML) is increasingly being applied in animal welfare monitoring to enhance the efficiency and accuracy of welfare assessments. Recent advances in remote sensing, computer vision and artificial intelligence (AI) have facilitated the development of new technologies for livestock biometrics, which extract key physiological parameters associated with animal welfare^13^. Automated monitoring systems, which include the use of sensors and ML algorithms, can provide continuous and non-invasive observation of animal behavior and health indicators^14^. This approach shows promising results and can be seen as a step toward automated health monitoring in farm animals^15^. Similar uses of ML/AI algorithms have been shown to be sensitive markers of health in some mouse models^8^.

**Fig. 1:**
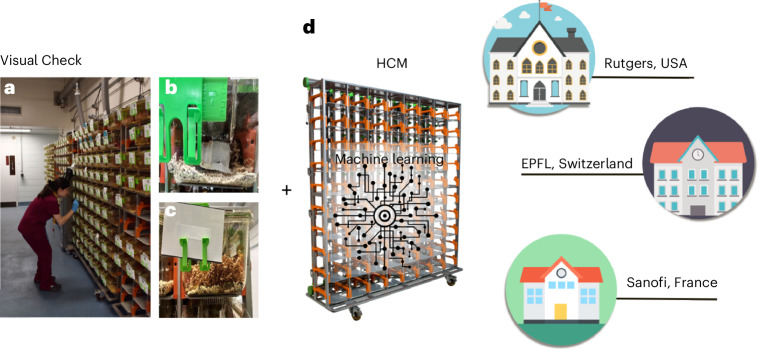
Representation of a daily health check performed by an animal caretaker, technology used and the study sites. **a**, An image of a daily check at Rutgers University. **b**,**c**, Enrichment and nest-obstructing view of the technician. **b**, The enrichment bag. Sometimes mice hide their babies inside the bag. **c**, The mice have opened the bag, taken out the strands of nesting material and built a nest inside which they burrow. **d**, DVC rack used and the three study sites. Canva was used to generate cartoons depicting the three sites.

Our study was conducted in three facilities across Europe and the USA (Fig. 1) that use similar HCM technologies, namely the Digital Ventilated Cages (DVC) that track continuous animal locomotion. The three institutions have different husbandry practices, and we retrospectively analyzed data collected by HCM over a period of 1–3 years with more than 100 cages per facility. We used capacitance-based sensor technology to monitor animal locomotion. Our hypothesis was that analysis of locomotion data using ML algorithms, combined with the ability of predictive AI to process this information and send alerts to staff, would identify animal distress earlier than visual health checks and thereby enhance early humane endpoint detection and improve animal welfare. This study seeks to validate the broader applicability of scalable HCM systems to revolutionize laboratory animal health monitoring, align with global regulatory standards and promote the ethical treatment of research animals.

## Results

An example of how the health checks are performed in the three facilities is shown in Fig. 1. Technicians at Rutgers University (Fig. 1a) use a pen light to assess whether animals are in distress; however, animals are often sleeping in their nests or enrichment devices, which reduces visibility (Fig. 1b,c). This is particularly problematic as removing the cages from the rack to check the animals is not encouraged at Rutgers and Ecole Polytechnique Fédérale de Lausanne (EPFL), as it can affect the animals’ sleep patterns and the rigor and reproducibility of research. Time-in-motion studies at Rutgers University showed that technicians typically spend between 4 and 8 s per cage during daily health checks.

**Fig. 2:**
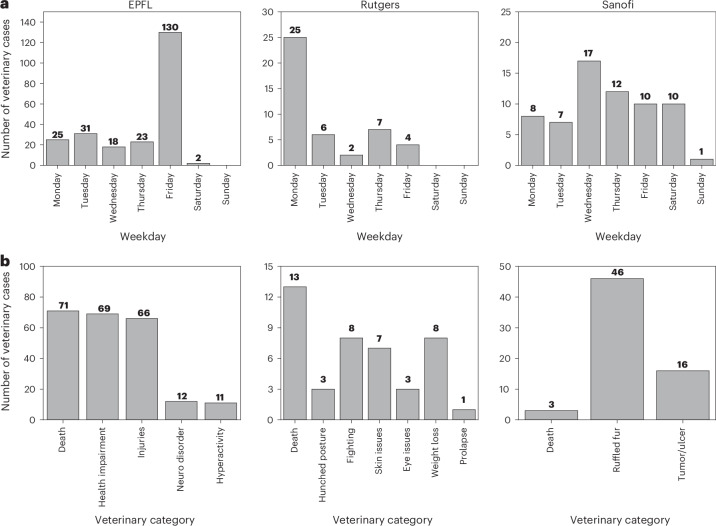
Distribution of veterinary cases by weekday and veterinary category across the three institutions. **a**, The number of verified clinical veterinary cases reported on a given weekday. **b**, The number of clinical cases per veterinary category at each of the study sites. The total number of cases for each category is given on top of the bars in the graphs.

To determine the efficiency of the daily health checks across the three institutions, we performed a retrospective analysis of all the clinical cases reported in the past 11 months (at Rutgers University, USA), 3 years (at EPFL, Switzerland) and 10 months (at Sanofi, France) from animals housed on the DVC rack. Our analysis shows that the highest number of clinical cases across all three institutions coincided with the cage change days EPFL (Friday), Rutgers (Monday) and Sanofi (Wednesday) (Fig. 2a) with a steep drop-off on weekends especially at Rutgers and EPFL. The drop observed on weekends at these two facilities may be attributed to the reduced number of staff present, who must check a much higher volume of cages than on regular workdays. In addition, weekend health checks tend to focus more on the presence of food and water and on whether any animals require veterinary treatment. The difference in clinical case detection between cage change and other days at Sanofi was not substantial. This could be because these were tumor studies, and the cages were removed from the rack daily for examination without needing to open them. The total number of confirmed clinical cases during the study period at the three institutions were EPFL (229), Rutgers (42) and Sanofi (65), categorized as shown in Fig. 2b. The density of animals in a cage and distribution of veterinary cases is shown in Supplementary Figs. 1 and 2, respectively. These data confirm that the brief visual checks for health are not very effective at detecting distress in animals. Most clinical cases are detected only when technicians have the opportunity and time to handle the animals and check them for signs of disease or distress during cage change.

**Fig. 3:**
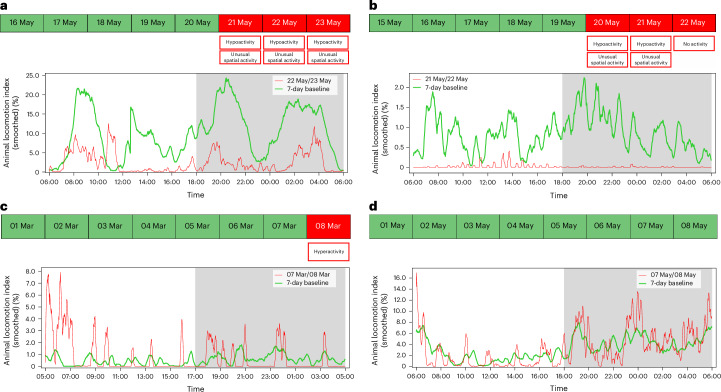
Example of graphical output from the welfare-check task generated by the algorithm. **a**–**d**, The green line indicates the average locomotor activity of the cage in the past 7 days. The red line displays the average activity of the cage in the past 24 h. In the background, the white and gray areas represent daytime and nighttime, respectively. Each panel includes a header bar indicating time progression in days, and the red blocks represent alerts triggered when abnormalities were identified by the algorithm. Shown are an example of hypoactivity (**a**), a case of a single-housed dead mouse (**b**) and examples of hyperactivity (**c**) and regular activity (**d**).

To improve the health checks, we developed ML/AI algorithms that analyze locomotor activity at night when mice are typically more active. The algorithm generates a pattern of activity for each cage over a 12-h period during the dark cycle. It then compares this pattern with the average activity in the same cage over the previous 7 days. The types of alerts generated by the algorithm are illustrated in Fig. 3. The activity for the day of the alert is shown with red lines, while the average activity from the past 7 days is shown with green lines. In addition, a visual indicator bar at the top of the graph signals whether the activity is normal (green bar) or abnormal (red bar with text for hypoactivity, hyperactivity or unusual spatial activity). Figure 3a shows an animal that has hypoactivity, while Fig. 3b shows no activity, suggesting a dead animal. Figure 3c reveals an activity pattern with several higher-than-normal peaks indicating hyperactivity. Figure 3d illustrates a normal activity pattern resulting in no additional tasks or alerts being raised. These visual prompts are used to prompt the technician to do a more thorough examination of the animals for signs of disease or distress.

An advantage of the proposed algorithm is that users can establish thresholds for detecting anomalies in the locomotion patterns, including the detection of true positives (TP) and false positives (FP). We used three ensemble models to analyze data from the three study sites, aiming to identify the threshold with the greatest potential for broader implementation across an entire facility. The maximum FP thresholds were set at 5%, 15% and 30% for models 1, 2 and 3, respectively. This flexibility allows a facility to establish limits that strike a balance between operational efficiency and the deployment of digital technology. An excessive number of false alerts has the potential to cause ‘user fatigue’, leading to a loss of confidence in the technology and thereby undermining its effectiveness.

The analysis was performed over a total of 1,367 cages from the three sites over distinct study periods (approximately 300 days for both Rutgers and Sanofi, and over 1,000 days at EPFL), resulting in 90,540 unique cage evaluations at EPFL, 51,094 at Rutgers and 15,367 at Sanofi, respectively (Table 1). In addition, Table 1 presents the rate of FP case detection using the three models. The retrospective starting point for the analysis was the date a verified veterinary clinical case was registered in each animal facility (day 0). As expected from the threshold limits set for the three models, the FP rate increased progressively from model 1 to model 3, with notable site-specific differences. The percentages at EPFL were more consistent with the algorithm performance obtained by the optimization process. Interestingly, Rutgers showed much smaller differences in false positives between models 1 and 2, probably due to the higher number of wild-type B6 mice in the cohort. Both Sanofi and Rutgers showed minimal differences between model 2 and model 3 (Table 1). This difference between the sites could be due to the lower number of cages and shortened assessment duration at Rutgers and Sanofi.

**Table 1:** Performance of the AI algorithms across the three institutions

				FPs		
Site	Number of unique cages	Number of days	Number of evaluations	Model 1	Model 2	Model 3
EPFL	374	1,382	91,610	6%	18%	28%
Rutgers	588	322	51,094	4%	9%	9%
Sanofi	405	288	15,367	6%	21%	23%

**Fig. 4:**
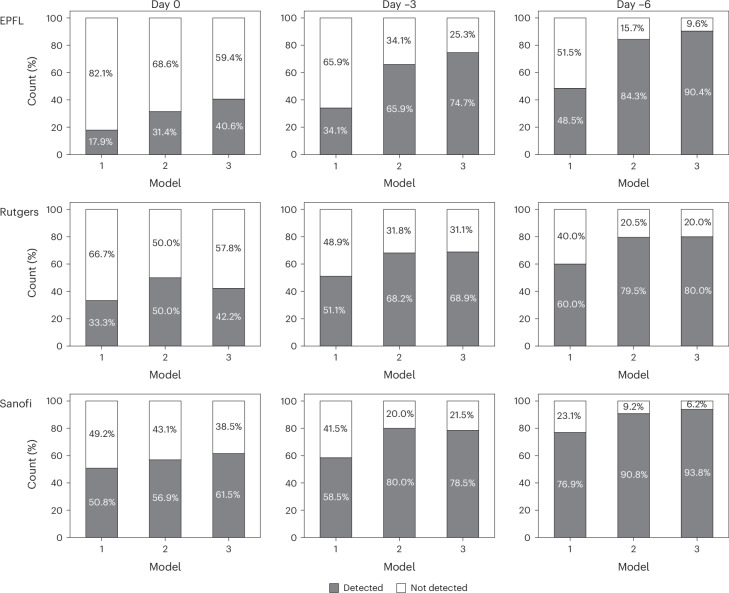
Detection rates of three ensemble models across three time intervals. The percentage of detected and not detected veterinary cases is shown over three time intervals: day 0 (when the clinical case was reported), from day 0 to –3 days and from day 0 to −6 days.

The rate of clinical case or TP detection on day 0 increased from model 1 to model 3 (Fig. 4). The increase in detection rate from model 1 to model 3 at each institution was as follows: EPFL increased from 17.9% (model 1) to 40.6% (model 3), and Sanofi increased from 50.8% (model 1) to 61.5% (model 3). However, at Rutgers University, model 3 showed a lower detection rate (42.2%) compared with model 2 (50%). Statistically significant differences between model 1 and model 2 were seen in all facilities and time intervals (McNemar test, *P* < 0.05, power between 0.71 and 1), with a minimal increase only at Sanofi (+6.1%). We expanded the analysis window to −3 and −6 days before day 0, to account for the fact that operator-reported cases are often biased toward certain weekdays and counted a detection as a TP if it happened within that window. This shows an increase in TP case detection when allowing for a wider detection window, for all three models (Fig. 4). A statistically significant difference between model 2 and model 3 was found only at EPFL, where the detection rate increased by 7–9 percentage points, whether the time interval was −3 or −6 days (McNemar test, *P* < 0.05, but power was limited (0.05–0.24) because of the small number of discordant pairs). Based on these results, we chose model 2 to present the remaining results in the report, as it provided the right balance between operational efficiency and TP detection efficiency. Results from models 1 and 3 are shown in Supplementary Figs. 3–6.

**Fig. 5:**
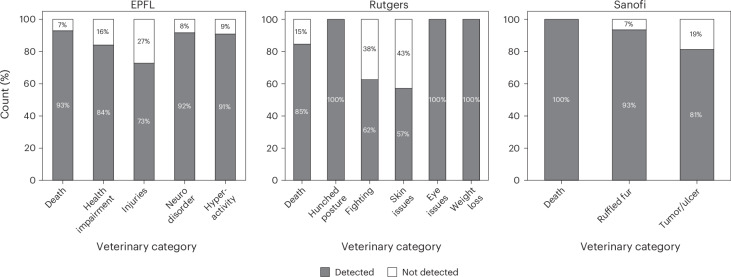
Detection rates of TP veterinary case categories using model 2. The percentage of cases detected for each category of veterinary cases is shown from day 0 to −6 days using ensemble model 2.

Figure 5 illustrates the category of TP clinical issues detected using model 2 from the three sites on day 0. The nomenclature for the clinical cases is different for the three institutions based on the available clinical records. Each clinical category was counted only if there were a minimum of three clinical cases. The algorithm detected 93% of found dead cases in EPFL (out of 71 cases), 85% at Rutgers (out of 13 cases) and 100% at Sanofi (out of 3 cases). The small percentage of undetected fatal cases at Rutgers and EPFL involved group-housed mice. The technology at this present time cannot track individual animal locomotor activity and thereby probably does not have the higher level of sensitivity to detect a 100% of the cases of spontaneous death. At Rutgers, the algorithm identified issues with hunched posture, eye issues and weight loss at 100% accuracy. Detection rates above 80% were also observed for health impairment, neurological disorders and hyperactivity at EPFL, as well as for ruffled fur issues and tumor or ulcers at Sanofi. The lowest percentages were observed for injuries at EPFL (73%) and fighting and skin issues at Rutgers (62% and 57%, respectively).

**Fig. 6:**
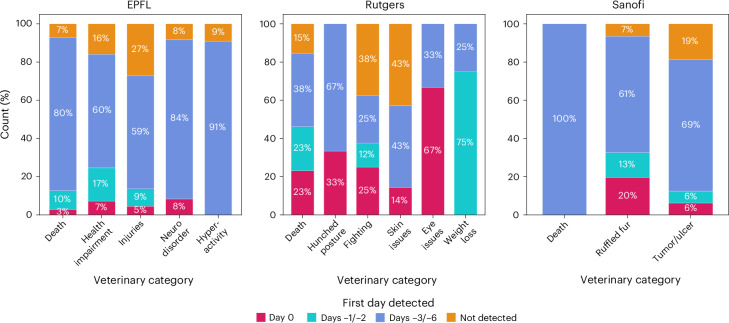
Temporal distribution of the first alerts generated by model 2 from day 0 to –6 days for each veterinary case category. The percentage of veterinary cases detected by ensemble model 2 is illustrated for the first time on day 0, between days −1 and −2 and between days −3 and –6.

To check the ability of the algorithm to detect subclinical cases from the TP subset, we expanded the analysis window to −3 and −6 days. Figure 6 shows when the algorithm detected anomalous behavior for the first time. In general, first detections of anomalies occurred between day −3 and −6, suggesting an early indication of the clinical issue. At EPFL and Sanofi, the TP detection was between 59% and 100% depending on the clinical categories on days −3 to −6. At Rutgers, some TP cases with skin issues (43%), fighting (38%) and ocular (67%) were not detected in earlier days. Earlier stages of these health issues might not have affected the locomotor activity of mice and thereby could have led to them not being detected by the algorithm.

## Discussion

Our study introduces an approach to welfare monitoring in laboratory mice by using continuous HCM using sensors underneath the cage combined with ML algorithms. This method addresses several limitations of traditional visual health checks, which are typically performed during the daytime and are often impeded by the presence of enrichment devices and the nocturnal behavior of mice^7,16^. Subclinical disease signs are missed using this approach. Visual observations are subject to subjective assessment and individual interpretation, which can reduce the effectiveness of daily welfare checks. Mice are prey species and will generally mask signs of pain^7,16^. This can result in subtle signs of distress being missed during the brief visual observation. Our results show that most clinical cases across the three sites are reported during cage-change days. During a cage change, the animal care technicians open the cage and handle the animals, increasing the chance of detecting abnormalities in their physical condition and signs of pain or distress. Opening cages or handling animals daily is not practical during routine health observations, as it can disrupt rodents’ sleep cycles, cause unnecessary stress that may confound experimental results, and require substantial staffing resources, particularly in large facilities housing thousands of mouse cages.

The importance of this study lies in its potential to markedly improve the accuracy and efficiency of health assessments in laboratory animals, thereby enhancing animal welfare and the quality of research data. Multiple published studies support the use of digital tools and AI algorithms in animal welfare monitoring^17–19^. AI has increasingly been used to estimate animal welfare or health indicators by analyzing images or videos^8,20^. AI systems can enable veterinarians to perform tasks more efficiently while providing new insights for the management and treatment of disorders, potentially translating to a better quality of life for animals and caregivers^21^. However, AI systems must be carefully designed to avoid bias and ensure fair and accurate evaluations^22^.

The continuous HCM system utilized in our study tracks locomotor activity in a non-invasive manner. A prior retrospective study of video monitoring data showed the utility of animal locomotion as a sensitive marker for predicting health in mouse models of oncology, respiratory and kidney disease^8^. The system with sensors described in this study generates substantially less volume of data compared with video monitoring systems, making it more adaptable for large-scale implementation in animal facilities. An important advantage of this technology is that the presence of enrichment inside the cage has no effect on the activity detection, unlike video-based technologies. An important aspect of this study is that the clinical cases were derived from a heterogeneous population spanning multiple research projects and institutions with differing husbandry practices and clinical reporting procedures. These results indicate that ML-based locomotion analysis can be applied as a tool to detect welfare issues in mice regardless of animal phenotype or institutional practices, and is therefore broadly applicable across laboratory animal facilities. Our results show that locomotion data analyzed by ML models can detect TP and FP clinical cases with a high degree of accuracy across all three institutions despite these differences in practices and without modifying clinical cases to fit a predefined scoring system, which validates the effectiveness of the technology.

The advantages of this study are the ability to continuously monitor animal activity without disrupting natural behaviors, the reduction of subjective bias inherent in human visual checks, and the provision of early alerts for potential health issues. Implementing a specific welfare algorithm that alerts animal caretakers, veterinarians, facility managers and scientists based on HCM data complements traditional health checks and enhances the traceability of welfare reporting. Early detection of impending clinical issues can help the veterinary staff to work with the research staff to define better end points, develop mitigation strategies or plan early euthanasia to collect valuable samples. Another advantage of the continuous assessment of locomotor activity is that we can obtain data on the possible disruption of mouse circadian rhythm. Sleep disruption, reduced activity or responsiveness during normally active periods, and increased activity during normally inactive periods can be indicators of adverse effects^23,24^ and can affect the rigor and reproducibility of research, particularly in neurobehavioral studies. These alerts can be used by researchers to either postpone behavior assessments or re-evaluate their experimental paradigm.

While the results are promising, some limitations exist in our study. The current version of the welfare algorithm did not detect a small percentage (9–20%) of verified clinical cases. The power of predictive AI is dependent upon the availability of large datasets to train/learn and make accurate predictions by identifying patterns and/or trends in data. Even though the dataset from the three institutions was reasonably large, more robust datasets are needed to increase the accuracy of the algorithm. As this technology is more widely implemented within the three facilities, we will have larger and more diverse datasets that will improve accuracy and reveal nuanced trends that are not apparent with smaller datasets. Another consideration for wide-scale implementation of the technology is that, while the ML models analyze data in real time, welfare alerts from the software are not reported to animal caretakers until the morning. This delay can pose ethical challenges regarding timely intervention for animals in distress. While these data are promising, the sample size of clinical cases for each category is too small to draw conclusions on specific patterns of detection. Also, it is very difficult to interpret the data across the sites owing to the different categories in which the clinical cases were recorded. It should be noted that the Night Welfare Check (NWC) alerts prompt an animal caretaker to do a more thorough physical examination of the animals in the cage compared with the fleeting 4–8-s visual check from outside the cage. This more detailed physical examination will help detect and confirm potential health issues regardless of how a particular institution might classify the clinical cases.

Compared with other ML approaches, such as fully supervised models that require large, labeled datasets, deep learning models that can capture complex patterns but are often difficult to interpret, or unsupervised methods that detect anomalies without any annotated data, this welfare-check algorithm uses a hybrid approach. By comparing metrics with historical baselines and using a genetic algorithm—a problem-solving method that starts with a population of random candidate solutions and iteratively evolves them over multiple generations to identify the best-performing ones—for optimization, the NWC algorithm provides a good balance between generalizability, performance and interpretability. Future improvements will include integrating daytime activity and 24-h circadian pattern analysis into the models, while accounting for the challenge of possible daytime disturbances due to procedures in the facility. In addition, more advanced models, such as neural networks, could be explored to identify complex patterns such as stereotypy, fighting-like behavior, depression or anxiety-like behaviors. In addition, integrating experimental data such as expected phenotypes or study endpoints into the user-verified information could allow the algorithm to learn and thereby predict accurate endpoints. A potential limitation of this algorithm is that it currently measures only locomotor activity, and not all clinical conditions necessarily affect locomotion. Another deficiency in the technology described is its inability to track individual mice when mice are group housed. Combining video technologies and radiofrequency identification tagging with the sensor boards could overcome these challenges. A scenario that could be highly beneficial would be the automatic generation of video clips whenever the sensor board detects abnormal locomotor activity. These video clips can then be either emailed or accessed remotely by the veterinary staff for review. This could enhance real-time detection of health issues and help the veterinary staff to intervene earlier.

Overall, our study demonstrates the potential of continuous HCM and ML/AI algorithms to transform welfare monitoring in laboratory mice. By providing continuous, non-invasive monitoring and early detection of health impairments, this approach addresses limitations of traditional visual health checks and enhances animal welfare. The implementation of such advanced monitoring system in rodent facilities can substantially improve the quality of research data and at the same time ensure the well-being of laboratory animals.

## Methods

The retrospective study was conducted on locomotion data collected from mice housed at three different sites (Rutgers University in the USA, Sanofi in France and EPFL in Switzerland) that utilize the DVC technology for housing animals to evaluate the performance of the welfare-check algorithm (Fig. 1). This study did not involve dedicated animals but relied only on data collected from animals enrolled in projects previously approved by the Institutional Animal Care and Use Committee (Rutgers), French Ministry for Higher Education and Research (Sanofi) and Cantonal Veterinary Office of the Canton of Vaud, Switzerland (EPFL), respectively. Mice were housed under a 12:12 light–dark cycle at 22 ± 2 °C, with relative humidity maintained at 55% ± 15%. Air changes in the DVC rack were set to 75 air changes per hour (ACH) per the manufacturer’s recommendations. All facilities were specific pathogen-free and excluded pathogens listed on the Federation of European Laboratory Animal Science Associations (FELASA) exclusion panel and, in the case of Rutgers, additional internal criteria. These included mouse parvovirus, minute virus of mice, mouse hepatitis virus, Theiler’s murine encephalomyelitis virus, murine rotavirus, Sendai virus, pneumonia virus of mice, reovirus, *Mycoplasma pulmonis*, *Corynebacterium bovis*, lymphocytic choriomeningitis virus, adenovirus, ectromelia virus, polyoma virus, Hantaan virus, murine chapparvovirus/mouse kidney parvovirus, mites and pinworms.

At Rutgers, mice were sourced either locally from faculty researchers or from an approved vendor (The Jackson Laboratory). Most of the mice were either C57BL/6J mice or transgenic mice on the B6 background. The transgenic mice were single-housed for phenotyping studies, while the B6 mice used for husbandry studies were housed in different configurations (one to five mice per cage). The cages were bedded with 350 ml of 1/8-inch pelleted cellulose bedding (Biofresh Performance Bedding, BioFresh), and each cage received crinkle paper nesting material as enrichment. Single-housed mice received a small enclosure as a secondary form of enrichment (Mouse Igloo, Bio-Serv). Cage changes were done based on the wetness of the bedding using a digital measure—Bedding Status Index. Mice were provided extruded 5058 diet (PicoLab Mouse Diet 20, LabDiet) ad libitum.

At EPFL, Switzerland, 71 different mouse lines were housed in DVC racks from May 2020 to February 2024. Mice were housed from one to five animals per cage with ad libitum access to food (SAFE 150) and acidified water. Each cage contained bedding (Tapvei, aspen bedding) and environmental enrichment including a cardboard tunnel, red plastic house, wood stick and nesting materials. Experimental cages were changed once a week, mainly on Fridays.

At Sanofi, France, a cohort of 684 C17 female SCID mice (CB17*/lCR-Prkd*c^scid^/lcrIcoCrl) was sourced from Charles River Laboratories, and eight NXG female mice (NOD-*Prkdc*^scid^-IL2rg^Tm1^/Rj) from Janvier Labs CERJ. Mice were monitored from March to December 2023. The mice were initially housed in standard individually ventilated cages upon arrival at ages 6–8 weeks and then transferred to the DVC rack following tumor implantation at ages 10–30 weeks. The mice were kept in groups ranging from two to six in these experimental cages, with constant access to food (SAFE R03) and water (filtered and autoclaved). Each cage was lined with aspen irradiated sawdust (Souralit, C24) and included enrichment elements such as a cardboard tunnel. Cage changes were performed weekly, mainly on Wednesdays.

The DVC (Tecniplast S.p.A.) system is an automated capacitance-based HCM technology that enables scalable and automatic data collection from multiple cages concurrently. Each cage has an electronic sensing board located beneath it, containing 12 contactless electrodes. These electrodes measure changes in electrical capacitance caused by animal movements, providing a non-intrusive method to record activity data^11^. This system facilitates continuous 24/7 recording of activity and offers an opportunity to assess the welfare of animals. A specialized ML algorithm, the NWC algorithm, was developed to analyze the data collected from these sensors and using predictive AI identify distressed animals by detecting anomalies and changes over time in their activity patterns. The algorithm evaluates animal behavior in each cage daily by analyzing metrics from the dark phase (nighttime) and generates tasks for inspecting cages with abnormal activity patterns. The computational requirements for the algorithm are minimal. The algorithm runs in the Amazon Web Services cloud and therefore does not demand any dedicated local hardware or information technology infrastructure from the facility. Unlike video-based systems that require high-bandwidth data transfer and intensive computing power (for example, graphics processing units), the NWC algorithm processes structured capacitance data, which are efficient to store and analyze. For example, in a facility with several thousand registered cages, the full analysis can be completed in less than 30 min, providing an individual outcome and chart for each cage. This architecture allows the algorithm to scale efficiently while keeping the computational burden invisible to the user and without affecting local operations.

From the raw signals (4 Hz), features were extracted to capture global activity (average of activations across time and electrodes^11^), spatial distribution of electrode activations^16^, temporal activity distribution (activity across different nighttime windows) and the positioning of active centroids (distances and areas defined by activated electrodes). For each metric, the algorithm constructs statistical baselines for each cage using historical data spanning 7 days to establish standard locomotion patterns and classifies cages as anomalous if deviations from the baselines are identified. The ML algorithm uses an ensemble approach, combining multiple metrics through a voting mechanism: if a specified number of metrics substantially deviate from their baselines, the algorithm identifies the cage as anomalous. The flowchart of the algorithm is shown in Supplementary Fig. 1.

Using a metaheuristic optimization technique derived from genetic algorithms^25,26^, the training of the algorithm underwent an iterative refinement process to determine the optimal combinations of metrics and vote thresholds (ensemble model) that detect substantial health issues with minimal false alerts. For the optimization, we used a dataset different from the one analyzed in the present study. The dataset included 817 cages without clinical signs and 218 cages with clinical signs documented by veterinary staff, each monitored over a period of 28 days, resulting in more than 28,000 daily evaluations. This dataset was split into training, validation and test sets to develop three ensemble models under different tolerated FP constraints (5%, 15% and 30% alerts generated on a reportedly normal animal). In the present study, we then applied these three models to data collected from three independent sites to assess their performance. Analyses were carried out using Python (Python Software Foundation) to process and visualize data, and R (R Foundation for Statistical Computing) to statistically evaluate performance differences between different ensemble models by conducting pairwise comparisons with the McNemar test, using a significance level of *α* = 0.05. The McNemar test was used because the model output is a dichotomous variable (‘detected’ or ‘not detected’).

### Reporting summary

Further information on research design is available in the Nature Portfolio Reporting Summary linked to this article.

## Online content

Any methods, additional references, Nature Portfolio reporting summaries, source data, extended data, supplementary information, acknowledgements, peer review information; details of author contributions and competing interests; and statements of data and code availability are available at 10.1038/s41684-026-01745-2.

## Supplementary information

Supplementary Information Supplementary Figs. 1–5.

Reporting Summary

## Data Availability

Aggregated data from the analysis will be available upon reasonable request to the corresponding author.

## References

[1] Miller, M. & Brielmeier, M. Environmental samples make soiled bedding sentinels dispensable for hygienic monitoring of IVC-reared mouse colonies. *Lab. Anim.* **52**, 233–239 (2018). 29145766 10.1177/0023677217739329

[2] National Research Council, Committee for the Update of the Guide for the Care and Use of Laboratory Animals, Institute for Laboratory Animal Research & National Academies Press in *Guide for the Care and Use of Laboratory Animals* xxv, 220, 112 (National Academies Press, 2011).

[3] *Animal Welfare Act and Animal Welfare Regulations* (US Department of Agriculture, Animal and Plant Health Inspection Service, 2005).

[4] *Directive 2010/63/EU of the European Parliament and of the Council of 22 September 2010 on the Protection of Animals Used for Scientific Purposes* Article 9,41 (Council of Europe, 2010).

[5] *Code of Practice for Monitoring the Welfare of Laboratory Animals* (Univ. Groningen, 2007).

[6] FELASA working group on revision of guidelines for health monitoring of rodents and rabbits et al. FELASA recommendations for the health monitoring of mouse, rat, hamster, guinea pig and rabbit colonies in breeding and experimental units. *Lab. Anim.* **48**, 178–192 (2014). 24496575 10.1177/0023677213516312

[7] Burkholder, T., Foltz, C., Karlsson, E., Linton, C. G. & Smith, J. M. Health evaluation of experimental laboratory mice. *Curr. Protoc. Mouse Biol.* **2**, 145–165 (2012). 22822473 10.1002/9780470942390.mo110217 PMC3399545

[8] Do, J. P. et al. Automated and continuous monitoring of animal welfare through digital alerting. *Comp. Med.* **70**, 313–327 (2020). 32414427 10.30802/AALAS-CM-19-000090 PMC7446638

[9] Bains, R. S. et al. Assessing mouse behaviour throughout the light/dark cycle using automated in-cage analysis tools. *J. Neurosci. Methods* **300**, 37–47 (2018). 28456660 10.1016/j.jneumeth.2017.04.014 PMC5909039

[10] Richardson, C. A. The power of automated behavioural homecage technologies in characterizing disease progression in laboratory mice: a review. *Appl. Anim. Behav. Sci.* **163**, 19–27 (2015).

[11] Iannello, F. Non-intrusive high throughput automated data collection from the home cage. *Heliyon* **5**, e01454 (2019). 30997429 10.1016/j.heliyon.2019.e01454 PMC6451168

[12] Ahloy-Dallaire, J., Klein, J. D., Davis, J. K. & Garner, J. P. Automated monitoring of mouse feeding and body weight for continuous health assessment. *Lab. Anim.* **53**, 342–351 (2019). 30286683 10.1177/0023677218797974

[13] Fuentes, S., Gonzalez Viejo, C., Tongson, E. & Dunshea, F. R. The livestock farming digital transformation: implementation of new and emerging technologies using artificial intelligence. *Anim. Health Res. Rev.* **23**, 59–71 (2022). 35676797 10.1017/S1466252321000177

[14] Ferdinandy, B. et al. Challenges of machine learning model validation using correlated behaviour data: evaluation of cross-validation strategies and accuracy measures. *PLoS ONE* **15**, e0236092 (2020). 32687528 10.1371/journal.pone.0236092 PMC7371169

[15] Sturm, V. et al. Combination of sensor data and health monitoring for early detection of subclinical ketosis in dairy cows. *Sensors* **20**, 1484 (2020). 32182701 10.3390/s20051484 PMC7085771

[16] Marx, J. O., Brice, A. K., Boston, R. C. & Smith, A. L. Incidence rates of spontaneous disease in laboratory mice used at a large biomedical research institution. *J. Am. Assoc. Lab. Anim. Sci.* **52**, 782–791 (2013). 24351767 PMC3838613

[17] Mosch, L. et al. Towards substitution of invasive telemetry: an integrated home cage concept for unobtrusive monitoring of objective physiological parameters in rodents. *PLoS ONE* **18**, e0286230 (2023). 37676867 10.1371/journal.pone.0286230 PMC10484458

[18] Vagima, Y. et al. Group activity of mice in communal home cage used as an indicator of disease progression and rate of recovery: effects of LPS and influenza virus. *Life Sci.* **258**, 118214 (2020). 32768585 10.1016/j.lfs.2020.118214

[19] Hasriadi, Dasuni Wasana, P. W., Vajragupta, O., Rojsitthisak, P. & Towiwat, P. Automated home-cage monitoring as a potential measure of sickness behaviors and pain-like behaviors in LPS-treated mice. *PLoS ONE* **16**, e0256706 (2021). 34449819 10.1371/journal.pone.0256706 PMC8396795

[20] Guo, Y., He, D. & Chai, L. A machine vision-based method for monitoring scene-interactive behaviors of dairy calf. *Animals* **10**, 190 (2020). 31978962 10.3390/ani10020190 PMC7071125

[21] Basran, P. S. & Appleby, R. B. The unmet potential of artificial intelligence in veterinary medicine. *Am. J. Vet. Res.* **83**, 385–392 (2022). 35353711 10.2460/ajvr.22.03.0038

[22] Liptovszky, M. Advancing zoo animal welfare through data science: scaling up continuous improvement efforts. *Front. Vet. Sci.* **11**, 1313182 (2024). 38298448 10.3389/fvets.2024.1313182 PMC10827962

[23] Abou-Ismail, U., Burman, O. H. P., Nicol, C. & Mendl, M. Can sleep behaviour be used as an indicator of stress in group-housed rats (*Rattus norvegicus*)? *Anim. Welf.* **16**, 185–188 (2007).

[24] Patti, C. L. et al. Effects of sleep deprivation on memory in mice: role of state-dependent learning. *Sleep* **33**, 1669–1679 (2010). 21120129 10.1093/sleep/33.12.1669 PMC2982737

[25] Holland, J. H. *Adaptation in Natural and Artificial Systems: An Introductory Analysis to Biology, Control and Artifical Intelligence* (MIT Press, 1992).

[26] Goldberg, D. E. *Genetic Algorithms* (Pearson Education India, 2013).

