# Supplementary material for: Enhanced health evaluation in mice using continuous home-cage monitoring and machine learning: a multicentric study
Source: Lab Anim (NY). 2026 May 28;55(7):275–81. doi: 10.1038/s41684-026-01745-2 (PMC13318627; doi:10.1038/s41684-026-01745-2)

lab animal

Supplementary information

https://doi.org/10.1038/s41684-026-01745-2

Enhanced health evaluation in mice using continuous home-cage monitoring and machine learning: a multicentric study

In the format provided by the authors and unedited

Figure S1. Distribution of veterinary cases by cage density across three institutions. Each bar represents the number of veterinary cases reported for cages with a given cage density.

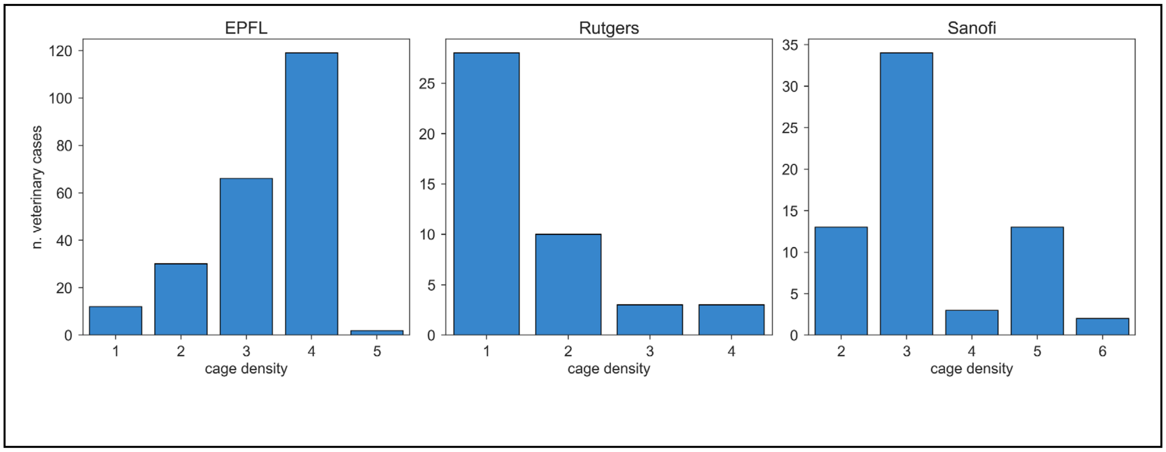

Figure S2. Detection rates of Model 1 across veterinary case categories. The figure shows the detection rates for each category of veterinary cases, using ensemble Model 1, and considering data from 6 days prior to and including day 0.

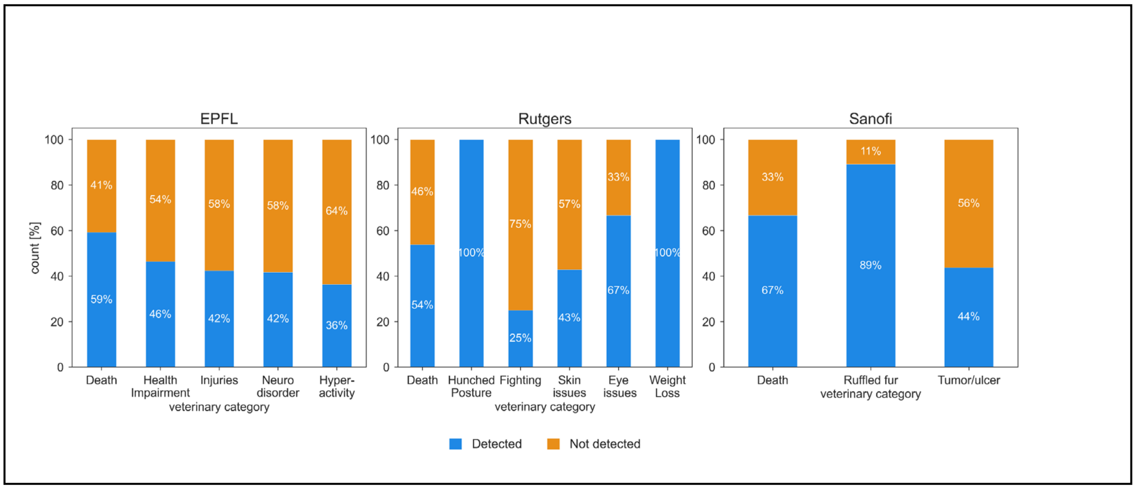

Figure S3. Detection rates of Model 3 across veterinary case categories. The figure shows the detection rates for each category of veterinary cases, using ensemble Model 3, and considering data from 6 days prior to and including day 0.

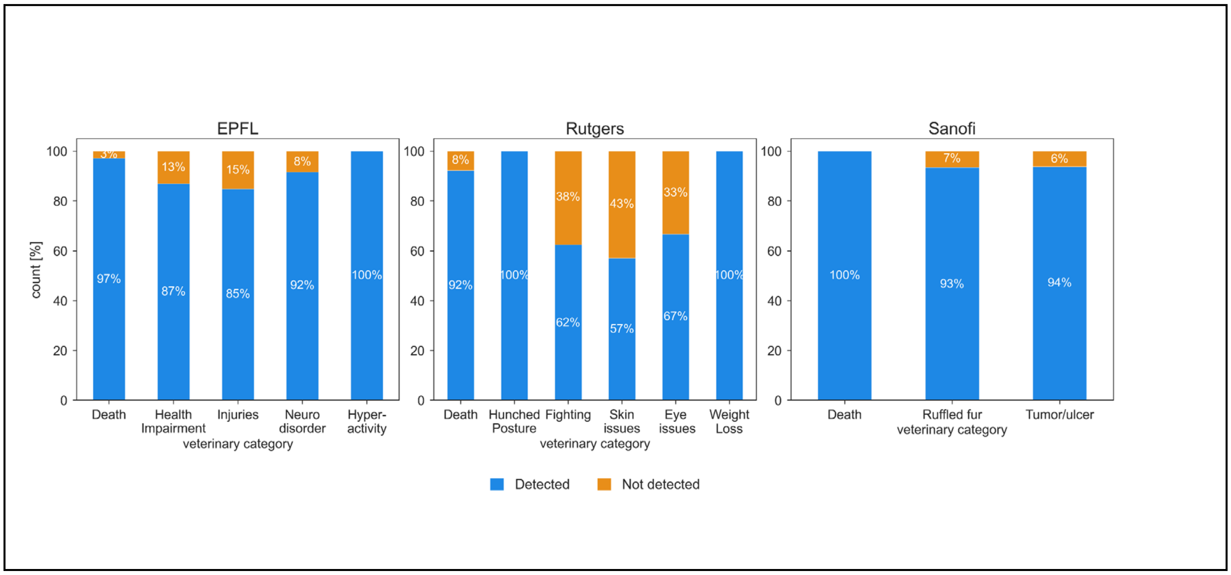

Figure S4. Detection rates of Model 1 across TP veterinary cases over 6 days. The figure illustrates the percentage of veterinary cases detected by Model 1 for the first time on day 0, or between days -1 to -2, and to -3 to -6, or not detected within the 6-days period. This allows to evaluate the potential early detection of clinical issues, for each veterinary category.

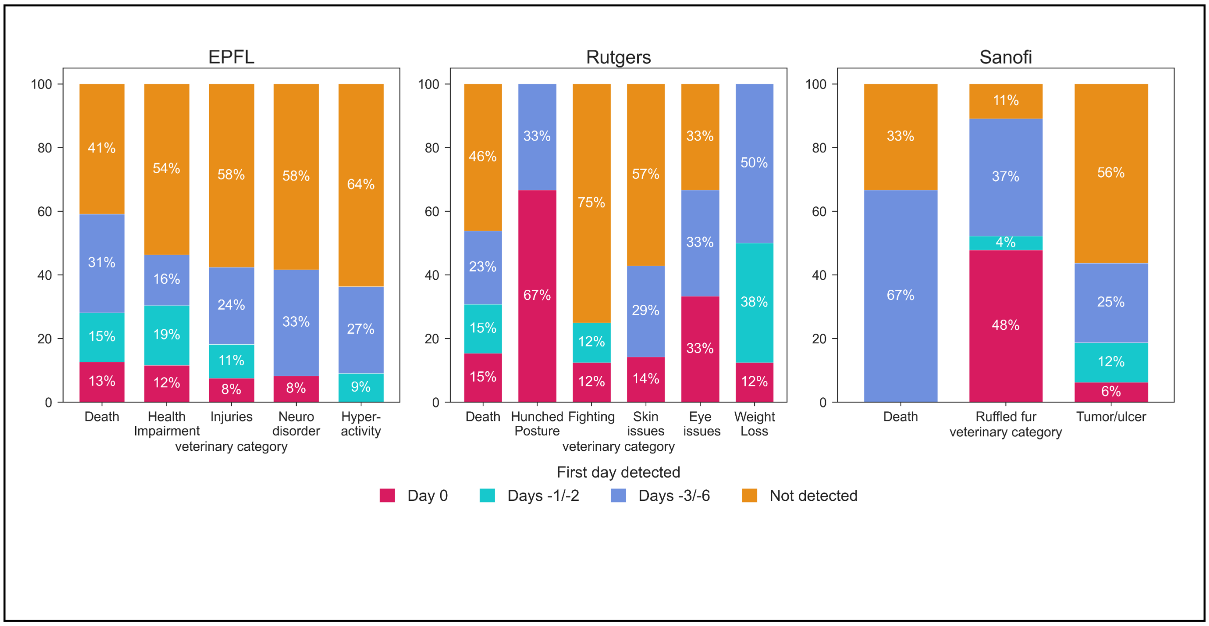

Figure S5. Detection rates of Model 3 across TP veterinary cases over 6 days. The figure illustrates the percentage of veterinary cases detected by Model 3 for the first time on day 0, or between days -1 to -2, and to -3 to -6, or not detected within the 6-days period. This allows to evaluate the potential early detection of clinical issues, for each veterinary category.

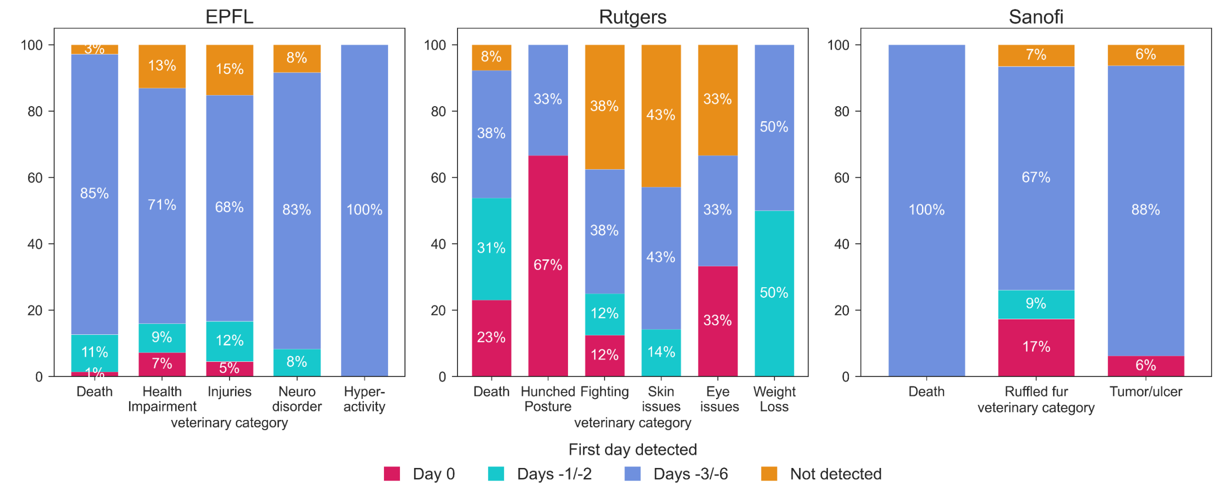

Supplement: Supplementary file 1 — Supplementary Figs. 1–5. [file 41684_2026_1745_MOESM1_ESM.pdf]
